# Call for Decision Support for Electrocardiographic Alarm Administration Among Neonatal Intensive Care Unit Staff: Multicenter, Cross-Sectional Survey

**DOI:** 10.2196/60944

**Published:** 2024-12-20

**Authors:** Xiaoli Tang, Xiaochen Yang, Jiajun Yuan, Jie Yang, Qian Jin, Hanting Zhang, Liebin Zhao, Weiwei Guo

**Affiliations:** 1 Shanghai Engineering Research Center of Intelligence Pediatrics Shanghai Children's Medical Center, School of Medicine Shanghai Jiao Tong University Shanghai China; 2 Neonatology Department Shanghai Children’s Medical Center, National Children’s Medical Center School of Medicine, Shanghai Jiao Tong University Shanghai China; 3 Child Health Advocacy Institute China Hospital Development Institute Shanghai China; 4 Shanghai Jiao Tong University School of Nursing Shanghai China; 5 Neonatology Department Nanfang Hospital Southern Medical University Guangzhou China; 6 Neonatal Respiratory Group of Chinese Physicians Association Guangzhou China; 7 Administrative Office Shanghai Children's Medical Center, School of Medicine Shanghai Jiao Tong University Shanghai China

**Keywords:** ECG alarm, electrocardiographic, perception, practice, decision-making, neonatal intensive care unit, health care providers, cross-sectional survey, nationwide

## Abstract

**Background:**

Previous studies have shown that electrocardiographic (ECG) alarms have high sensitivity and low specificity, have underreported adverse events, and may cause neonatal intensive care unit (NICU) staff fatigue or alarm ignoring. Moreover, prolonged noise stimuli in hospitalized neonates can disrupt neonatal development.

**Objective:**

The aim of the study is to conduct a nationwide, multicenter, large-sample cross-sectional survey to identify current practices and investigate the decision-making requirements of health care providers regarding ECG alarms.

**Methods:**

We conducted a nationwide, cross-sectional survey of NICU staff working in grade III level A hospitals in 27 Chinese provinces to investigate current clinical practices, perceptions, decision-making processes, and decision-support requirements for clinical ECG alarms. A comparative analysis was conducted on the results using the chi-square, Kruskal-Wallis, or Mann-Whitney *U* tests.

**Results:**

In total, 1019 respondents participated in this study. NICU staff reported experiencing a significant number of nuisance alarms and negative perceptions as well as practices regarding ECG alarms. Compared to nurses, physicians had more negative perceptions. Individuals with higher education levels and job titles had more negative perceptions of alarm systems than those with lower education levels and job titles. The mean difficulty score for decision-making about ECG alarms was 2.96 (SD 0.27) of 5. A total of 62.32% (n=635) respondents reported difficulty in resetting or modifying alarm parameters. Intelligent module–assisted decision support systems were perceived as the most popular form of decision support.

**Conclusions:**

This study highlights the negative perceptions and strong decision-making requirements of NICU staff related to ECG alarm handling. Health care policy makers must draw attention to the decision-making requirements and provide adequate decision support in different forms.

## Introduction

Neonatal intensive care units (NICUs) are clinical settings equipped with over 40 types of medical devices with alarm functions [1,2]. Several studies have reported that alarm problems associated with electrocardiographic (ECG) monitors were the most prominent among all devices with high sensitivity and low specificity in clinical settings [3]. An observational study by Cho et al [4] revealed that ECG alarms accounted for 81.9% of the total 2184 alarms, far more than the number of alarms triggered by other devices. Li et al [5] found that each ECG monitors in the NICU generated over 177 alarms daily, of which over 95% were deemed false alarms. It is worth noting that several other studies have shown that existing clinical management strategies are insufficient in enhancing the precision of ECG alarms and providing clinical staff with effective decision support related to ECG alarms [6-9].

The prolonged exposure of health care providers to “high sensitivity and low specificity” alarms will disrupt their work and lead to emotional and work-related stress [3]. The alarms can also cause alarm fatigue and ignoring of signals in health care providers [10], which may increase health risks for patients or even death [11]. According to a national survey, up to 57.1% of adverse events were related to vital signs [12]. The Joint Commission published 98 cases of alarm-related accidents over a 3-year period, including 80 deaths and 13 cases of permanent loss of function. Meanwhile, it is worth noting that this result only represented less than 10% of the hospitals in the actual occurrence of alarm-related hazards [13].

In addition, sound exposure from frequent alarms can also have negative effects on neonatal outcomes, including growth and development. Due to the neonates’ immature systems, prolonged noise stimuli can not only disrupt the development of the auditory cortex, leading to hearing loss [14], but can also imbalance the balance of excitation and inhibition in the cerebral cortex, causing neurological disorders and affecting brain development [15]. The American Academy of Pediatrics recommends that sound exposure in the NICU should not exceed 45 dB [16]. However, the noise levels in NICU wards can be up to 70 dB in general, with a maximum noise of up to 100 dB [17-19], which is significantly beyond the international threshold [20]. Zhang et al [20] further measured the sources of noise in the NICU and found that the ECG monitor was the instrument with the highest alarm volume (55.7-86.3 dB) in the NICU setting. Thus, the problem of ECG alarms in NICUs is closely related to the safety and health of newborns.

The Joint Commission’s newly released Patient Safety Goals for 2022 included the safe use of device alarms as one of its goals [21]. The Chinese Hospital Association’s Patient Safety Goals released in 2019 also specified the need to “enhance medical device safety and alarm management” [22]. The relevant system evaluation pointed out that false alarms of ECG monitors in NICU are mainly caused by irregular alarm management behaviors such as irrational setting of alarm values and parameters and answering or delayed answering, which depends on whether health care providers can make scientific decisions and self-management during the alarm setting and after they have occurred [9]. Decision-making behavior refers to the complex process of making a decision based on cognition, personal preferences, values, and other trade-offs when confronted with a problem; however, to the best of our knowledge, there has been no study investigating decision-making with regard to the ECG alarm. On the other hand, although a few studies have reported that alarm improvement management programs using alarms, algorithmic optimization software, and clinical decision support systems (CDSSs) [23] can promote decision-making about alarms, decrease false alarms [9,24-26], reduce alarm fatigue [27,28], and enhance alarm response speeds [29-31], these studies had limited sample numbers, and it was difficult to extend their results. Additionally, no studies have assessed the clinical implementation of ECG alarm practices [32].

Therefore, the objective of this study was to conduct a comprehensive nationwide, multicenter, large-sample cross-sectional survey to identify the current practices and investigate the decision-making requirements of health care providers regarding ECG alarms. The findings of this study serve as a solid foundation for enhancing decision support and developing strategies that address the challenges associated with ECG alarms.

## Methods

### Study Design and Participants

This was a strategic research and consulting project launched by the Respiratory Group of the Neonatologists’ Branch of the Chinese Medical Doctors Association, China. The inclusion criteria were as follows: (1) the number of ECG monitors in the NICU was ≥50, (2) on-the-job registered physicians and nurses, and (3) years of work experience ≥3 years. The exclusion criterion was medical staff in training. This study adhered to the STROBE (Strengthening the Reporting of Observational Studies in Epidemiology) reporting guidelines, ensuring transparent and comprehensive reporting of the research findings.

### Ethical Considerations

Prior to the commencement of the study, ethics approval was obtained from the ethics committee of Shanghai Children’s Medical Center (approval SCMCIRB-YJ2022001). This study strictly adhered to ethical guidelines, with all participants signing informed consent forms to ensure their awareness of the research objectives and procedures. All data were anonymized to protect participant privacy.

### Sampling Method

**Figure 1:**
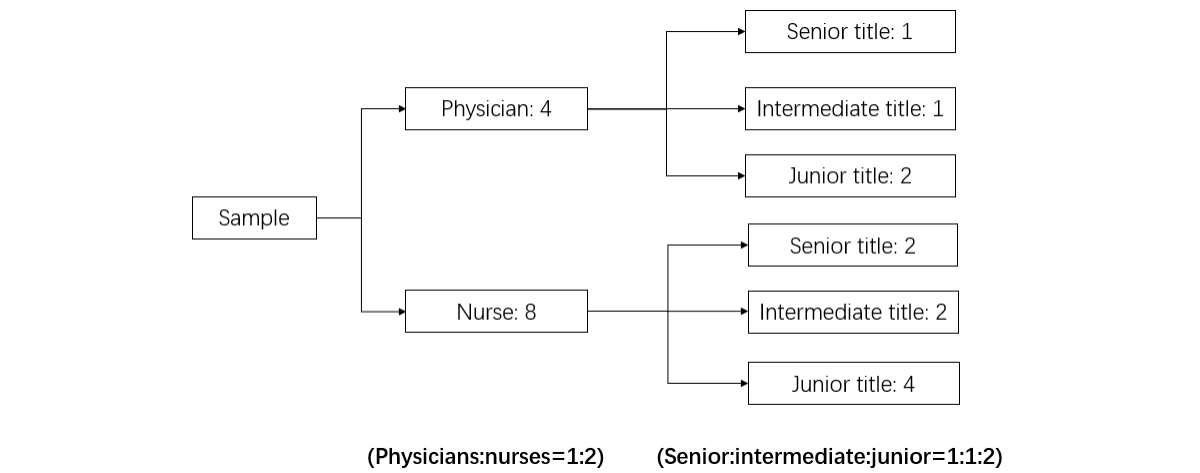
Sampling method.

We first screened all hospitals within the Chinese Physicians Association, which encompasses the majority of hospitals with NICUs nationwide, to identify those that met the inclusion criteria. In each NICU, an equal number of health care providers were surveyed. An anonymous web-based questionnaire was administered to physicians and nurses in a 1:2 ratio, that is, the numbers of physicians and nurses selected from each NICU were 4 and 8, respectively, with a total of 12 respondents. Both physician and nurse subgroups were selected by convenience sampling according to a 1:1:2 ratio of professional titles (senior, intermediate, and junior titles) [33] (Figure 1).

### Data Collection

We used the Wenjuanxing website, a secure web-based platform designed to capture data from web-based investigations. This platform was used to create a survey project located on a static URL that could be remotely accessed via a smartphone browser regardless of the geographical position of the proxy. There was a web-based informed consent page for obtaining e-consent prior to the web-based study questionnaire page, and consent questions were added to the page in yes or no format. On the first page of the questionnaire, the aims, objectives, instructions, and questions were designed to be mandatory, so participants who did not complete all the questions received a warning before submission. Therefore, all the collected questionnaires were completed without missing data. The questionnaire response time was approximately 10 minutes. According to the sampling method mentioned earlier, the link to the e-questionnaire was distributed to the responsible personnel at each selected hospital via email. Those who expressed willingness to participate were provided with a web-based anonymous questionnaire by the designated person. The platform’s settings ensured that multiple entries from the same individual were prevented by tracking unique IP addresses.

### Measurements

#### Sociodemographic Information

Sociodemographic characteristics included sex, age, educational level, marital status, hospital, job title, profession, position, and years of work experience. The job-title classification system is widely acknowledged and implemented throughout the Chinese medicine and health care system, ranking medical professionals as junior, intermediate, and senior. Physicians and nurses can advance to their respective titles if they meet the necessary conditions, including educational requirements, clinical competency criteria, and passing the National Uniform Title Examination.

#### Survey Questionnaire Regarding Clinical Alarms on Medical Devices (Chinese Version)

The original questionnaire was initially administered by the Healthcare Technology Foundation in 2005 and was revised in 2011 [34] and 2016 [35]. In 2023, the 2016 version was translated from English to Chinese by Wang [36]. The questionnaire was designed to assess perceptions and practices of medical staff regarding clinical alarms across 7 dimensions (nuisance alarms, experience of alarm systems, alarm notifications, smart alarms, hospital policies and procedures, alarm management improvements, and alarm-related adverse events). It contained 31 items, of which 16 items consisted of agreement levels on alarms and alarm management. These were rated on a 5-point Likert scale (1=strongly disagree to 5=strongly agree), with higher scores indicating stronger agreement with the item statements. Items 6, 7, and 9 in dimension II were reverse-scored. In addition, 8 items concerned practices related to clinical alarms (response options including “no,” “yes,” and “not sure”). Each item was analyzed based on the proportion of respondents selecting each option, with a higher proportion indicating that the practice was more commonly used. The remaining 7 items were open-ended questions for each dimension that were used to collect the respondent’s subjective opinion (see Part II in Multimedia Appendix 1). The survey showed sound internal consistency with a Cronbach α of 0.766, an item content validity index ranging from 0.86 to 1.00, and a scale content validity index of 0.98 [36]. The questionnaire exhibited good construct validity.

#### Questionnaire on Decision-Making and Decision-Support Regarding ECG Alarms

A questionnaire was developed to assess the decision-making processes and decision-support requirements of medical staff regarding clinical alarms. First, we created an initial version of the questionnaire by developing items based on the self-evaluation section conducted by He et al [37]. Second, an expert panel comprising 2 medical scientists, 2 senior nurses, and a data scientist was consulted to refine these items. Third, 3 doctors and 7 nurses took a pretest of the questionnaire, and modifications were made according to their feedback to obtain the final version of the questionnaire.

The final questionnaire contained the following three sections:

1. Confidence and difficulty of decision-making: This section included 8 items, each rated on a 5-point Likert scale (1=low and 5=high). Higher scores indicated that the health care providers had greater confidence or more difficulty in their decision-making process.
2. Decision-making basis: This section included 3 items. The first item investigated the basis for decision-making and included 9 multiple-choice items. A higher proportion indicated that the basis for making decisions about ECG alarms was more commonly used. The second and third items were designed to evaluate the effectiveness and accessibility of the existing decision-making bases, respectively, which were rated on a 5-point Likert scale. Higher scores indicated that the basis was perceived as more effective or accessible.
3. Decision support needs: This section included 6 items, aimed at exploring the preferred forms and specific functions of decision support. Items 12 and 13 were multiple-choice questions regarding the preferred forms by health care providers. The remaining items were rated on a 5-point Likert scale, with higher scores indicating stronger agreement with the specific function requirements.

### Statistical Analysis

According to our pilot study, the mean confidence and difficulty score for the decision-making about ECG alarms were 4.04 (SD 0.51) and 2.88 (SD 0.40) of 5, respectively. A sample size of 570 and 920 will produce a 2-sided 95% CI with a distance from the mean to the limits that is less than or equal to 0.04 if the population SD is estimated to be 0.4 and 0.51 by a previous sample of size 30, respectively. Considering around a 5% dropout rate, the expected sample size in this study was 969 participants. Descriptive statistics were used to describe the respondents’ sociodemographic information as well as their responses to the questions. A comparison analysis was conducted to test whether the perception differed by educational level, job title, and profession using a chi-square test, Kruskal-Wallis test, or Mann-Whitney *U* test. All statistical analyses in this study were 2-tailed, a *P* value of less than .05 was considered statistically significant, and all analyses were carried out using SPSS statistical software (version 20.0; IBM Corp).

## Results

### General Information

**Figure 2:**
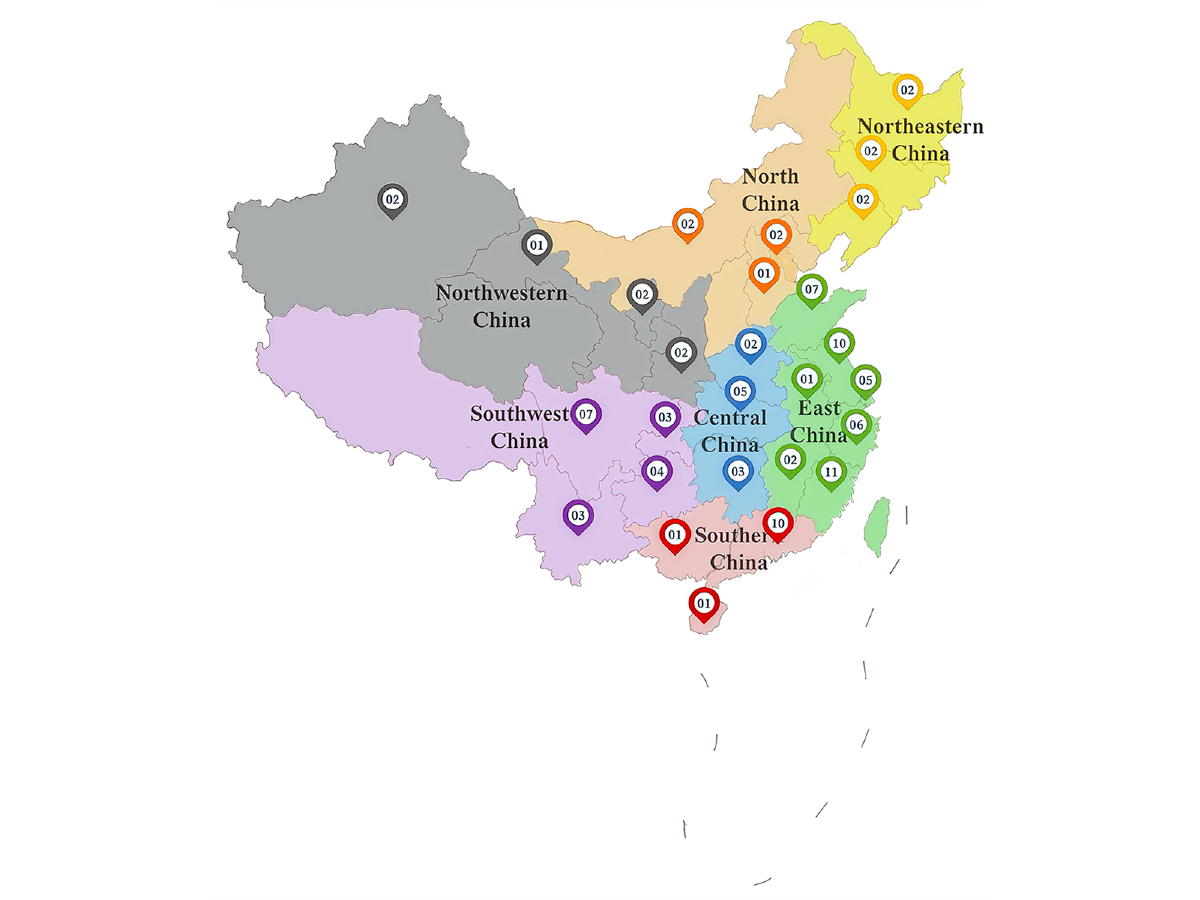
Geographical region distribution of the hospitals.

In this cross-sectional study, data were collected through a web-based survey conducted between October 1, 2023, and December 30, 2023. The survey aimed to cover nearly all geographical regions of China, and the percentages of sample size distributed in each region are detailed in Table 1. The respondents were from 99 hospitals representing 27 province-level regions in China (Figure 2). Finally, 1019 respondents were included in the data analysis with a response rate of 85.77%. All respondents worked in grade III level A hospitals. The detailed demographic characteristics of the respondents are presented in Table 1.

**Table 1:** Demographic characteristics of the respondents (N=1019).

Characteristics			Values
**Sex, n (%)**			
	Male	105 (10.30)	
	Female	914 (89.70)	
**Education background, n (%)**			
	Associate degree or below	94 (9.23)	
	Bachelor degree	674 (66.14)	
	Master degree or above	251 (24.63)	
**Marital status, n (%)**			
	Single	238 (23.36)	
	Married	763 (74.87)	
	Other	18 (1.77)	
**Area of the hospital, n (%)**			
	East China	415 (40.73)	
	North China	70 (6.87)	
	Northeastern China	70 (6.87)	
	Southern China	108 (10.60)	
	Northwestern China	85 (8.34)	
	Southwest China	153 (15.01)	
	Central China	108 (10.60)	
	Other	10 (0.98)	
**Type of hospital, n (%)**			
	General hospital	586 (57.51)	
	Specialized hospital	423 (41.51)	
	Other	10 (0.98)	
Age (years), mean (SD)			34.65 (6.93)
Children, mean (SD)			0.94 (0.76)
Length of work experience (years), mean (SD)			11.42 (7.44)
Length of work experience in intensive care unit (years), mean (SD)			8.53 (6.20)
Daily working hours, mean (SD)			8.96 (2.22)
**Profession, n (%)**			
	Nurse	666 (65.36)	
	Physician	287 (28.16)	
	Other	66 (6.48)	
**Position, n (%)**			
	Archiater	96 (9.42)	
	Physician-in-charge	141 (13.84)	
	In-training resident	50 (4.91)	
	Head nurse	70 (6.87)	
	Specialist nurse	138 (13.54)	
	Bedside nurse	458 (44.94)	
	Other	66 (6.48)	
**Job title, n (%)**			
	Senior	186 (18.25)	
	Intermediate	415 (40.73)	
	Junior or below	418 (41.02)	

### Perceptions and Practices Regarding Clinical ECG Alarms

Dimensions I and II indicate health care providers’ clinical negative perceptions related to ECG alarms. Additionally, dimensions III to VII indicate health care providers’ perceptions and practices of ECG alarm management initiatives. The detailed scores and the choice proportion from dimensions I to VII are found in Tables 2 and 3.

**Table 2:** Clinical perceptions related to electrocardiographic alarms (N=1019).

Dimension and item			Scores, mean (SD)		“Agree” or “strongly agree,” n (%)
**Dimension I: nuisance alarms**					
	2.1 Nuisance alarms occur frequently	3.00 (1.12)		391 (38.37)	
	2.2 Nuisance alarms disrupt patient care	3.11 (1.15)		513 (50.34)	
	2.3 Nuisance alarms reduce trust in alarms and cause caregivers to inappropriately turn alarms off at times other than setup or procedural events	2.32 (1.19)		230 (22.57)	
	Mean score for each item in dimension I	2.81 (0.90)		—^a^	
**Dimension II: experience of alarm systems**					
	2.5 Properly setting alarm parameters and alerts is overly complex in existing devices	2.53 (1.03)		221 (21.69)	
	2.6 Newer monitoring systems have solved most of the previous problems we experienced with clinical alarms	2.49 (0.93)		570 (55.94)	
	2.7 The alarms used on my floor/area of the hospital are adequate to alert staff of potential or actual changes in a patient’s condition	2.05 (0.80)		827 (81.16)	
	2.8 There have been frequent instances where alarms could not be heard and were missed	2.21 (0.97)		134 (13.15)	
	2.9 Clinical staff is sensitive to alarms and responds quickly	1.80 (0.66)		933 (91.56)	
	2.10 When a number of devices are used with a patient, it can be confusing to determine which device is in an alarm condition	2.53 (1.15)		274 (26.89)	
	2.11 Environmental background noise has interfered with alarm recognition	2.85 (1.15)		400 (39.25)	
	Mean score for each item in dimension II	2.35 (0.58)		—	
**Dimension III: alarm notification**					
	2.14 Alarm integration and communication systems are useful for improving alarm management and response	3.89 (0.77)		758 (74.39)	
	2.16 Central alarm management staff responsible for receiving alarm messages and alerting appropriate staff is helpful	3.85 (0.83)		730 (71.64)	
	Mean score for each item in dimension III	3.87 (0.69)		—	
**Dimension IV: smart alarms**					
	2.19 Smart alarms would be effective to use for reducing false alarms	3.75 (0.79)		648 (63.59)	
	2.20 Smart alarms would be effective to use for improving clinical response to important patient alarms	3.83 (0.76)		711 (69.77)	
	Mean score for each item in dimension IV	3.89 (0.73)		—	
**Dimension V: hospital policies and procedures**					
	2.24 Clinical policies and procedures regarding alarm management are effectively used in my facility	3.79 (0.81)		699 (68.60)	
**Dimension VII: alarm-related adverse events**					
	2.30 Since the implementation of the 2019 Patient Safety Goals issued by the Chinese Hospital Association regarding the alarm management program, your hospital has experienced a decrease in the occurrence of adverse events related to alarms	3.77 (0.75)		640 (62.81)	

^a^Not applicable.

**Table 3:** Clinical practices of electrocardiographic alarm management initiatives (N=1019).

Dimensions and items		Yes, n (%)	No, n (%)	Not sure, n (%)
**Dimension III: alarm notification**				
	2.13 Does your institution use alarm integration and communication systems (eg, pagers, cell phones, and other wireless devices) to notify alarms?	458 (44.95)	415 (40.73)	146 (14.33)
	2.15 Does your institution use “monitor watchers” in a central viewing area to observe and communicate alarm conditions to caregivers?	336 (32.97)	499 (48.97)	184 (18.06)
**Dimension IV: smart alarms**				
	2.18 Does your institution use Smart Alarm Systems?	368 (36.11)	407 (39.94)	244 (23.95)
**Dimension V: hospital policies and procedures**				
	2.22 Have you received education on the setup and operation of device alarms?	687 (67.42)	243 (23.85)	89 (8.73)
	2.23 Does your institution have a requirement to document that the alarms are set and are appropriate for each patient?	425 (41.71)	412 (40.43)	182 (17.86)
**Dimension VI: alarm management improvements**				
	2.26 Has your institution developed clinical alarm improvement initiatives over the past 2 years?	554 (54.37)	184 (18.06)	281 (27.58)
	2.27 Does your institution have a program in place to improve the security of alarm management?	647 (63.49)	142 (13.94)	230 (22.57)
**Dimension VII: alarm-related adverse events**				
	2.29 Has your institution experienced adverse patient events in the last 2 years related to clinical alarm problems?	171 (16.78)	576 (56.53)	272 (26.69)

### Different Personal Characteristics of Health Care Providers Toward Clinical Perceptions of ECG Alarms

#### Sex

Comparing female health care providers toward clinical perceptions of ECG alarms, male health care providers had more negative perceptions of experiences with alarm systems (*P*=.008). Female health care providers had more positive attitudes toward improving the current status of ECG alarms through adhering to hospital regulations (*P*<.001; Tables 4 and 5).

**Table 4:** Descriptive analysis of different personal characteristics of health care providers toward clinical perceptions of electrocardiographic alarms (N=1019).

Variables			Values, n (%)		Dimension I: nuisance alarms								Dimension II: experience of alarm systems						
					Mean (SD)		*t* test (*df*)			*P* value		Mean (SD)			*t* test (*df*)			*P* value	
**Sex**								0.16 (1017)		.87						2.67 (1017)			.008^a^
	Male	105 (10.30)		2.82 (0.91)							2.49 (0.56)								
	Female	914 (89.70)		2.81 (0.90)							2.34 (0.57)								
**Education background**								4.50 (2, 1016)^a^		.01^b^						11.84 (2, 1016)^a^			<.001
	Master degree or above	251 (24.63)		2.90 (0.87)							2.49 (0.54)								
	Bachelor degree	674 (66.14)		2.81 (0.91)							2.32 (0.57)								
	Associate degree or below	94 (9.22)		2.58 (0.93)							2.20 (0.62)								
**Job title**								4.61 (2, 1016)^a^		.01^b^						8.11 (2, 1016)^a^			<.001
	Senior	186 (18.25)		2.95 (0.87)							2.48 (0.53)								
	Intermediate	415 (40.73)		2.84 (0.90)							2.37 (0.58)								
	Junior or below	418 (41.02)		2.72 (0.92)							2.28 (0.59)								
**Profession**								–1.99 (951)		.047^b^						–5.59 (951)			<.001
	Nurse	666 (65.35)		2.80 (0.93)							2.29 (0.58)								
	Physician	287 (28.16)		2.92 (0.84)							2.52 (0.53)								

^a^*F* test.

^b^*P*<.05.

**Table 5:** Descriptive analysis of the electrocardiographic alarm management initiatives among health care providers with different personal characteristics (N=1019).

Variables		Values, n (%)	Dimension III: alarm notification			Dimension IV: smart alarms				Dimension V: hospital policies and procedures		
			Mean (SD)	*t* test (*df*)	*P* value	Mean (SD)	*t* test (*df*)	*P* value	Mean (SD)		*t* test (*df*)	*P* value
										
**Sex**				–0.81 (1017)	.42		–0.29 (1017)	.78			–3.74 (1017)	<.001
	Male	105 (10.30)	3.81 (0.63)			3.77 (0.70)			3.51 (0.82)			
	Female	914 (89.70)	3.88 (0.70)			3.79 (0.74)			3.82 (0.80)			
**Education background**				6.48 (2, 1016)^a^	.002^b^		1.91 (2, 1016)^a^	.15			12.81 (2, 1016)^a^	<.001
	Master degree or above	251 (24.63)	3.80 (0.68)			3.75 (0.70)			3.60 (0.77)			
	Bachelor degree	674 (66.14)	3.86 (0.69			3.78 (0.74			3.83 (0.81			
	Associate degree or below	94 (9.22)	4.10 (0.71)			3.92 (0.74)			4.04 (0.80)			
**Job title**				1.61 (2, 1016)^a^	.20		4.44 (2, 1016)^a^	.012^b^			7.43 (2, 1016)^a^	.001^b^
	Senior	186 (18.25)	3.84 (0.68)			3.67 (0.74)			3.62 (0.82)			
	Intermediate	415 (40.73)	3.84 (0.71)			3.76 (0.74)			3.77 (0.82)			
	Junior	418 (41.02)	3.92 (0.67)			3.86 (0.72)			3.89 (0.77)			
**Profession**				1.14 (951)	.26		1.46 (951)	.14			6.04 (567.42)	<.001
	Nurse	666 (65.36)	3.87 (0.71)			3.80 (0.76)			3.89 (0.81)			
	Physician	287 (28.16)	3.82 (0.65)			3.72 (0.69)			3.56 (0.77)			

^a^*F* test.

^b^*P*<.05.

#### Job Title and Education Level

Individuals with higher education levels (*P*=.01; *P*<.001) and job titles (*P*=.01; *P*<.001) had more negative perceptions of nuisance alarms and experiences with alarm systems than those with lower education levels and job titles (Table 4). In addition, individuals with lower job titles had more positive perceptions toward improving the current status of ECG alarms by adhering to hospital regulations and the application of smart alarms (*P*=.001; *P*=.012; Table 5).

#### Profession

Compared to the nurses, physicians had more negative perceptions with ECG monitors with regard to nuisance alarms and alarm systems (*P*=.047; *P*<.001). Additionally, nurses were more likely to agree that alarm management could be improved through policy implementation than physicians (*P*<.001).

### Difficulty and Confidence in Decision-Making About ECG Alarms

**Figure 3:**
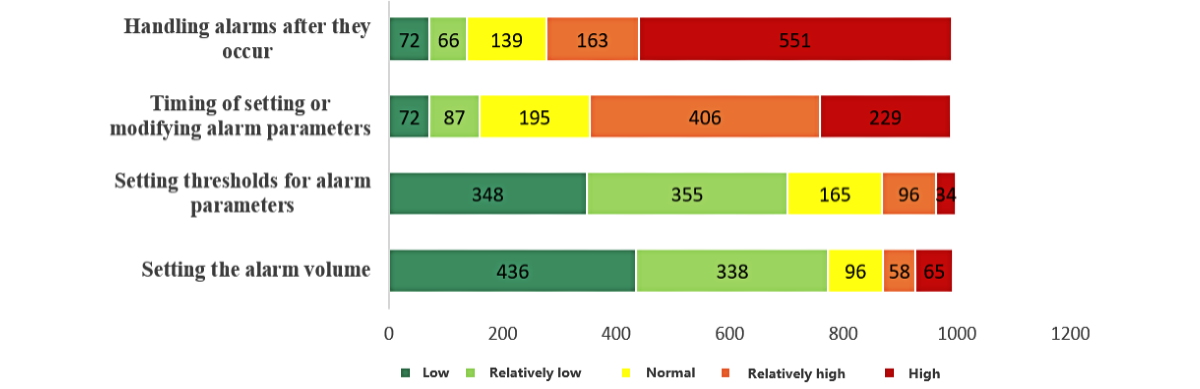
Difficulty of decision-making regarding electrocardiographic alarms.

In total, the mean confidence and difficulty scores for decision-making about ECG alarms were 3.99 (SD 0.63) and 2.96 (SD 0.27) of 5, respectively. Furthermore, 70.07% (n=714) of the respondents reported difficulty or great difficulty in the response and disposition alarms after they occurred, while 62.32% (n=635) reported difficulty in the timing of resetting or modifying alarm parameters (Figure 3). The rankings of confidence scores are in contrast to the difficulty scores, except for “response and disposition after an alarm occurs” (Table 6).

**Table 6:** Difficulty and confidence scores of decision-making about electrocardiographic alarms.

Items	Difficulty score, mean (SD)	“High” or “relatively high,” n (%)	Confidence score, mean (SD)	“High” or “relatively high,” n (%)
Response and disposition after an alarm occurs	4.06 (1.27)	714 (70.07)	4.06 (0.69)	870 (85.38)
Timing of resetting or modifying alarm parameters	3.64 (1.14)	635 (62.32)	3.88 (0.76)	761 (74.68)
Setting thresholds for alarm parameters	2.11 (1.09)	130 (12.76)	4.00 (0.71)	850 (83.41)
Setting the alarm volume	1.97 (1.17)	123 (12.07)	4.03 (0.74)	852 (83.61)
Mean score	2.96 (0.27)	—^a^	3.99 (0.63)	—

^a^Not applicable.

### Basis of Decision-Making Regarding ECG Alarms

Colleagues and leaders (n=891, 87.44%) were considered the most accessible decision-making bases, and clinical guidelines were considered the most effective (n=954, 93.62%). Table 7 shows the details of the selection of each decision-making basis.

**Table 7:** Accessibility and effectiveness scores of decision-making basis.

	Effectiveness score, mean (SD)	“Effective” or “very effective,” n (%)	Accessibility score, mean (SD)	“Accessible” or “very accessible,” n (%)
Primary study	3.99 (0.84)	776 (76.15)	3.73 (0.96)	641 (62.90)
Academic conference	4.06 (0.82)	817 (80.18)	3.75 (0.96)	660 (64.77)
Thematic training	4.08 (0.79)	831 (81.55)	3.76 (0.95)	663 (65.06)
Textbook	4.15 (0.81)	870 (85.38)	4.00 (0.92)	810 (79.49)
Colleagues and leaders	4.14 (0.76)	873 (85.67)	4.28 (0.75)	891 (87.44)
Systematic review	4.22 (0.74)	893 (87.63)	3.97 (0.87)	761 (74.68)
Standard operating procedure	4.30 (0.74)	924 (90.68)	4.15 (0.79)	858 (84.20)
Instruction book	4.37 (0.73)	925 (90.78)	4.26 (0.79)	885 (86.85)
Clinical guideline	4.42 (0.69)	954 (93.62)	4.21 (0.75)	884 (86.75)

### Needs for Decision Support in ECG Alarm Management

The preferred forms of decision support by health care providers are presented in Table 8. “Intelligent module–assisted decision support systems” was the most popular form of decision support. Further, Table 9 revealed specific function development regarding intelligent module–assisted decision support systems for ECG monitors.

**Table 8:** Forms of decision support for electrocardiographic monitors.

Items	Decision support forms on alarm settings, n (%)	Decision support forms on responding to and handling alarms after they occur, n (%)
Intelligent module–assisted decision support systems	722 (70.85)	710 (69.68)
Consultation with professionals	684 (67.12)	689 (67.62)
Knowledge repository	595 (58.39)	578 (56.72)
eBook	534 (52.40)	532 (52.21)
Paper-based book	462 (45.34)	442 (43.38)

**Table 9:** Specific function development for electrocardiographic (ECG) monitors.

Item	“Important” or “very important,” n (%)	Mean (SD)
Develop a standardized device interface to integrate ECG monitors with other devices	833 (81.75)	4.17 (0.78)
Intelligent decision support for alarm threshold setting for newborns of different birth gestational ages module	820 (80.47)	4.10 (0.79)
Optimizing algorithm models to achieve accurate alarms	814 (79.88)	4.09 (0.75)
Functional modules for grading alarms based on the severity of the patient’s condition	806 (79.10)	4.04 (0.76)

## Discussion

### Strengths

This was the first national-scale survey that aimed to examine clinical response and management regarding ECG alarms at both the practical and perceptual levels. The study covered more than 1019 medical staff members from 27 provinces in China, which is the largest sample size to date. The overall sample size was representative and large, which can help extend our results to reflect clinical practice as well as decision-making processes and needs related to ECG alarms. Overall, the study found that most departments have implemented initiatives for ECG alarm management. However, health care providers reported experiencing a significant number of nuisance alarms and held negative perceptions regarding alarms in clinical settings. Moreover, health care providers continue to face challenges in decision-making and accessing decision support for alarm settings and handling. These findings provide valuable insights for developing future strategies to enhance ECG alarm management.

### NICU Health Care Providers Have Alarm-Related Negative Perceptions and Practices

In this study, we found that more than half of the respondents reported that “nuisance alarms disrupt patient care,” and 38.37% (n=391) of them reported that “nuisance alarms occur frequently.” Altogether, 16.78% (n=171) of respondents reported that alarm-related adverse events related to ECG alarms still occurred in the past 2 years. These results show that there is currently a relative lack of initiative in the management and clinical response to ECG alarms. Most previous studies focused on nurses, with less attention paid to physicians’ perceptions and practices regarding ECG alarms [32,36,37]. Our study found that physicians had higher negative perceptions of ECG alarms than nurses.

In addition, we found that health care providers with higher education levels and job titles had more negative perceptions of nuisance alarms and experiences with alarm systems. This may be due to the fact that this population usually has a greater sense of responsibility and vigilance toward ECG alarms [24]. Interestingly, we found that health care providers with lower job titles had more positive attitudes toward improving the current status of ECG alarms through adhering to hospital regulations and the application of smart alarms but had fewer negative perceptions of nuisance alarms for ECG monitors than those with higher job titles. These 2 slightly contradictory results are thought-provoking. They suggested that health care providers with low job titles may be less vigilant about clinical alarms and may not pay as much attention to them, which may pose a hidden risk to clinical safety.

Moreover, in our study, the proportion of female health care providers was much higher than that of male health care providers, which was in line with the general characteristics of the sex ratio of medical staff in maternal and child hospitals in China [38,39]. We found that male health care providers had more negative perceptions regarding experiences with alarm systems, whereas female health care providers had more positive attitudes toward improving the current status of ECG alarms by adhering to hospital regulations. This might be because male health care providers tended to display more impatient and careless personality traits compared to female health care providers. These personality traits make male health care providers less likely to recognize alarm reminders and more prone to negative emotional reactions in their clinical work. In contrast, female health care providers generally demonstrate greater patience, which makes them more receptive to alarm management policies. However, the sample size of male participants included in this study was limited. Future research should investigate the perceptions of clinical practices among male health care providers in larger populations.

### NICU Health Care Providers Have Clinical Decision-Making Difficulties With ECG Monitor Parameter Settings

The effectiveness and accessibility of evidence have important impacts on the decision-making support for health care providers [40]. We found that although clinical guidelines were considered to be the most effective decision-making basis for ECG alarm parameter settings, 13.25% (n=135) of the respondents still had difficulties of availability in the clinical settings. It is worth noting that consulting colleagues and leaders remained the most convenient and accessible method for most health care providers, despite the reported limited effectiveness. Hence, the basis of decision-making should be provided in more friendly and convenient ways.

A meta-analysis by Shen and Xiong [41] showed that the parameter setting is an important factor influencing the occurrence of ECG alarm artifacts and poor response to alarms in neonates. This study revealed that decision-making difficulties related to ECG alarms have been significantly underestimated and inadequately addressed, particularly when it comes to setting or modifying the alarm parameters. In addition, we found that health care providers considered “responding to and handling alarms after they occur” to be the most difficult, but interestingly, they also had the highest level of confidence in it. This indicates that the current clinical training is effective in improving the competency of health care providers to respond to and manage alarms [42-44]. Accordingly, we hypothesized that the current clinical training has a limited effect on improving health care providers’ competency regarding “timing of setting or changing alarm parameters” and “setting thresholds for alarm parameters.”

On the other hand, it is worth noting that the ability to change alarm settings in a timely manner relies on the objective judgment of changes in the patient’s condition. This is highly dependent on the professional competence of health care providers, and it could be a fatal weakness especially in health providers with lower job titles. Hence, it is essential to create specialized decision-making support systems or improve decision-making procedures to facilitate this intricate process.

### High Demand for Intelligent Clinical Decision-Making Support Systems Among NICU Health Care Providers

Several guidelines and evidence summaries have included intelligent alarm management in their recommendations for ECG monitoring [26,45]. The CDSS uses artificial intelligence techniques to collect and analyze patient data from electronic medical records, based on established guidelines and high-quality clinical evidence [46]. This study showed that intelligent module–assisted decision support systems have become the most popular decision-making support method, and the selection rate is much higher than that of traditional decision applications.

Specific features should include categorizing alarms by priority, differentiating alarm notifications, and filtering unnecessary alarms [47]. In this study, we found that the highest demand was to “develop a standardized device interface to integrate ECG monitors with other devices.” This indicates that ECG monitors should be integrated with ventilators and other equipment to provide clinically meaningful alarms and to reduce the incidence of repeated alarms or alarm artifacts. Additionally, we found a high demand for the development of “intelligent decision support for alarm threshold settings for newborns of different birth gestational ages module.” This demand was even higher than that for “optimizing algorithm models to reduce alarm artifacts.” These results provide important evidence to provide sustainable technical solutions for clinical responses to ECG alarms.

### Limitations

This study had several limitations. First, all respondents volunteered to complete the questionnaires and may thus be subject to participant bias. Second, the data relied on self-reported measurements. Therefore, further research should be conducted to design observational studies or qualitative interviews that provide more robust evidence.

### Comparison With Prior Work

#### Evidence Before This Study

Alarm problems associated with ECG monitors were found to be the most prominent among all devices in the NICU. We conducted a comprehensive search of academic databases, including PubMed, Web of Science, Wanfang Data, CNKI, and China Science and Technology Journal Database, using specific keywords “electrocardio/ECG/EKG” and “NICU/ICU/intensive care” in combination with terms such as “clinical alarm/false alarm/alarm management/alarm improvement/alarm fatigue/alarm rejection/alarm apathy.” Our search was limited to original publications between January 1, 2020, and August 20, 2023. Previous studies have consistently demonstrated that ECG alarms in the NICU exhibit a characteristic pattern of “high sensitivity and low specificity.” Specifically, more than 95% of ECG alarms in this setting were identified as false alarms [5]. Prolonged exposure to such alarms can lead to alarm fatigue among health care providers, potentially resulting in disregarding important signals and an increased risk of adverse events for patients. Moreover, repeated exposure to alarm sounds can have detrimental effects on neonatal infants, including disruptions in the development of the auditory cortex, neurological disorders, and impairments in brain development. While previous studies have investigated the knowledge of clinical staff regarding ECG alarms, they have often neglected to examine their actual clinical practices. Consequently, the challenges and decision-making processes involved in ECG alarm management remain unclear.

#### Added Value of This Study

This was a cross-sectional survey covering most provinces and all geographical regions of China. To our knowledge, the study was the first national study to investigate both physicians and nurses, which provided reliable data for assessing practices and perceptions of clinical ECG alarms and summarized the related characteristics. It was also noted that decision-making difficulties and decision-support requirements of ECG alarms are greatly underrecognized and undertreated, especially for resetting or modifying alarm parameters. Additionally, intelligent module–assisted decision support systems have become the most popular form of decision support.

#### Implications of All the Available Evidence

The findings demonstrate that all NICU staff members reported negative experiences with ECG alarms, encompassing both clinical handling and management initiatives. Nevertheless, physicians had more negative perceptions toward ECG alarms than nurses, emphasizing the need for greater attention toward the physician population. Additionally, the study highlighted that over half of the NICU staff reported difficulties in making decisions related to resetting or modifying alarm parameters. There was a significant demand for the implementation of intelligent module–assisted decision support systems to address this challenge.

### Conclusions

This cross-sectional survey highlights the negative perceptions and practices as well as the strong decision-making requirements of NICU staff related to ECG alarm handling. Despite existing routine management measures, decision-making support related to ECG alarms remains a significant concern in the NICU. Intelligent CDSSs may become the most favored tool for overcoming the timing and threshold of ECG parameter settings.

## Appendix

Multimedia Appendix 1 A survey on the current clinical status of ECG alarm management among NICU healthcare providers.

## Abbreviations

CDSS: clinical decision support system
ECG: electrocardiographic
NICU: neonatal intensive care unit
STROBE: Strengthening the Reporting of Observational Studies in Epidemiology

## Data Availability

The datasets generated and analyzed during this study are not publicly available due to privacy and confidentiality concerns but are available from the corresponding author on reasonable request.
