# Correction: Call for Decision Support for Electrocardiographic Alarm Administration Among Neonatal Intensive Care Unit Staff: Multicenter, Cross-Sectional Survey

**DOI:** 10.2196/101652

**Published:** 2026-06-29

**Authors:** Xiaoli Tang, Xiaochen Yang, Jiajun Yuan, Jie Yang, Qian Jin, Hanting Zhang, Liebin Zhao, Weiwei Guo

**Affiliations:** 1 Shanghai Engineering Research Center of Intelligence Pediatrics, Shanghai Children's Medical Center, School of Medicine, Shanghai Jiao Tong University Shanghai China; 2 Neonatology Department, Shanghai Children’s Medical Center, National Children’s Medical Center, School of Medicine, Shanghai Jiao Tong University Shanghai China; 3 Child Health Advocacy Institute, China Hospital Development Institute Shanghai China; 4 Shanghai Jiao Tong University School of Nursing Shanghai China; 5 Neonatology Department, Nanfang Hospital, Southern Medical University Guangzhou China; 6 Neonatal Respiratory Group of Chinese Physicians Association Guangzhou China; 7 Administrative Office, Shanghai Children's Medical Center, School of Medicine, Shanghai Jiao Tong University Shanghai China

In “Call for Decision Support for Electrocardiographic Alarm Administration Among Neonatal Intensive Care Unit Staff: Multicenter, Cross-Sectional Survey” [1], the authors noted one error in the Ethical Considerations section. The ethics approval number was mistakenly stated as “SCMCIRB-K2024014-1.” The correct ethics approval number for the study reported in this article is “SCMCIRB-YJ2022001,” which was granted by the Ethics Committee of Shanghai Children’s Medical Center on November 22, 2022. This ethics approval was obtained for a project comprising a survey of neonatal intensive care unit electrocardiographic alarm status and alarm management, as well as the development of an intelligent monitoring device for high-risk neonates.

In the Ethical Considerations section, the following sentence was revised:

Prior to the commencement of the study, ethics approval was obtained from the ethics committee of Shanghai Children’s Medical Center (approval SCMCIRB-K2024014-1).

This sentence now reads:

Prior to the commencement of the study, ethics approval was obtained from the ethics committee of Shanghai Children’s Medical Center (approval SCMCIRB-YJ2022001).

The correction will appear in the online version of the paper on the JMIR Publications website, together with the publication of this correction notice. Because this was made after submission to PubMed, PubMed Central, and other full-text repositories, the corrected article has also been resubmitted to those repositories.
