# Supplementary material for: Call for Decision Support for Electrocardiographic Alarm Administration Among Neonatal Intensive Care Unit Staff: Multicenter, Cross-Sectional Survey
Source: J Med Internet Res. 2024 Dec 20;26:e60944. doi: 10.2196/60944 (PMC11699503; doi:10.2196/60944)
Supplement: Multimedia Appendix 1 [file jmir_v26i1e60944_app1.docx]

A survey on the current clinical status of ECG alarm management among NICU healthcare providers

Dear Healthcare Colleagues:

Thank you for participating in this survey. Before you begin, please note the following:

**Purpose:** We have been commissioned by the Chinese Nursing Association's Pediatrics Committee to conduct a clinical research study on the current clinical practices, perceptions, decision-making processes, and decision-support requirements for clinical electrocardiography (ECG) alarms among neonatal intensive care unit (NICU) healthcare providers. This survey consists of **3 parts**: a general information questionnaire, a survey questionnaire regarding clinical alarms on medical devices, and a questionnaire on decision-making and decision-support regarding ECG alarms.

**Voluntary Participation:** Your participation in this survey is entirely voluntary. You may choose to withdraw at any point without any consequences.

**Confidentiality:** Your responses will be kept confidential, and any data collected will be used solely for research purposes. Personal identifiers will not be linked to your responses.

**Risks and Benefits:** There are no significant risks associated with participating in this survey. However, your responses will contribute to important findings that may benefit NICU healthcare providers.

**Contact Information:** If you have any questions or concerns about this survey, please contact Xiaoli Tang (Tel：18930871509).

By proceeding with this survey, you acknowledge that you have read and understood this consent statement and agree to participate.

Please read this questionnaire in detail and fill it out carefully and truthfully according to the questions. Filling out the questionnaire takes up your valuable time, for which we express our sincere thanks!

**Part I General information**

1. Gender: □Male □Female
2. Education background

□Associate's degree or below

□Bachelor's degree

□Master's degree or above

1. Marital status

□Single

□Married

□Other

1. Age: **______**
2. Number of children: **______**
3. Length of work experience:  **______** year
4. Length of work experience in ICU: **______** year
5. Daily working hours: **______** H
6. Profession: □Nurse □Physician □Other
7. Position:

□Archiater

□Physician-in-charge

□Regulated physician

□Head nurse

□Specialist nurse

□Bedside nurse

□Other

1. Job title:

□Junior or below

□Intermediate

□Senior

1. Your hospital is: ________
2. Total number of beds in your hospital's neonatal ward: ________
3. Total number of beds in NICU: ________
4. Area of the hospital：

□East China

□North China

□Northeastern China

□Southern China

□Northwestern China

□Southwest China

□The Central of China

□Other

1. Type of hospital：______

□General hospital

□Specialized hospital

□Other

**Part II Survey questionnaire regarding clinical alarms on medical devices**

**[Introduction]:** This survey addresses the current status of clinical alert issues related to cardiac monitoring devices. The survey includes seven dimensions: nuisance alarms, alarm system experience, alarm notification, smart alarms, hospital requirements, alarm management improvement, and alarm-related adverse events. Nuisance alarms are alarms triggered by invalid events or alarms triggered by valid events that do not require clinical intervention. Please tick the appropriate option or fill in the box with your views according to your situation.

| **Dimension** | **Items** | **Self-Evaluation** | | | | |
| --- | --- | --- | --- | --- | --- | --- |
| **Dimension I Nuisance Alarms** | 1. Nuisance alarms occur frequently. | Strongly disagree | Disagree | Not sure | Agree | Strongly disagree |
|  | 2. Nuisance alarms disrupt patient care. | Strongly disagree | Disagree | Not sure | Agree | Strongly disagree |
|  | 3.Nuisance alarms reduce trust in alarms and cause caregivers to inappropriately turn alarms off at times other than setup or procedural events. | Strongly disagree | Disagree | Not sure | Agree | Strongly disagree |
|  | ***4. Please fill in your opinion on "Nuisance Alerts":*** | | | | | |
| **Dimension II Experience of Alarm Systems** | 5. Properly setting alarm parameters and alerts is overly complex in existing devices. | Strongly disagree | Disagree | Not sure | Agree | Strongly disagree |
|  | 6. Newer monitoring systems have solved most of the previous problems we experienced with clinical alarms. | Strongly disagree | Disagree | Not sure | Agree | Strongly disagree |
|  | 7. The alarms used on my floor/area of the hospital are adequate to alert staff of potential or actual changes in a patient’s condition. | Strongly disagree | Disagree | Not sure | Agree | Strongly disagree |
|  | 8. There have been frequent instances where alarms could not be heard and were missed. | Strongly disagree | Disagree | Not sure | Agree | Strongly disagree |
|  | 9. Clinical staff is sensitive to alarms and responds quickly. | Strongly disagree | Disagree | Not sure | Agree | Strongly disagree |
|  | 10. When a number of devices are used with a patient, it can be confusing to determine which device is in an alarm condition. | Strongly disagree | Disagree | Not sure | Agree | Strongly disagree |
|  | 11. Environmental background noise has interfered with alarm recognition. | Strongly disagree | Disagree | Not sure | Agree | Strongly disagree |
|  | ***12. Please fill in your opinion on the "Experience of Alarm Systems":*** | | | | | |
| **Dimension III Alarm Notification** | 13. Does your institution use alarm integration and communication systems (eg. pagers, cell phones, and other wireless devices) to notify alarms? | NO | Not sure | YES | — | — |
|  | 14. Alarm integration and communication systems are useful for improving alarms management and response. | Strongly disagree | Disagree | Not sure | Agree | Strongly disagree |
|  | 15.Does your institution use “monitor watchers" in a central viewing area to observe and communicate alarm conditions to caregivers? | NO | Not sure | YES | — | — |
|  | 16.Central alarm management staff responsible for receiving alarm messages and alerting appropriate staff is helpful. | Strongly disagree | Disagree | Not sure | Agree | Strongly disagree |
|  | ***17. Please fill in your opinion on "Alarm Notification":*** | | | | | |
| **Dimension IV Smart Alarms** | 18.Does your institution use Smart Alarm Systems? | NO | Not sure | YES | — | — |
|  | 19.Smart alarms would be effective to use for reducing false alarms. | Strongly disagree | Disagree | Not sure | Agree | Strongly disagree |
|  | 20.Smart alarms would be effective to use for improving clinical response to important patient alarms. | Strongly disagree | Disagree | Not sure | Agree | Strongly disagree |
|  | ***21. Please fill in your opinion about "*** ***Smart Alarms ":*** | | | | | |
| **Dimension V Hospital policies and procedures** | 22.Have you received education on the setup and operation of device alarms? | NO | Not sure | YES | — | — |
|  | 23. Does your institution has a requirement to document that the alarms are set and are appropriate for each patient? | NO | Not sure | YES | — | — |
|  | 24. Clinical policies and procedures regarding alarm management are effectively used in my facility. | Strongly disagree | Disagree | Not sure | Agree | Strongly disagree |
|  | ***25. Please fill in your opinion about the "*** ***Hospital policies and procedures ":*** | | | | | |
| **Dimension VI Alarm Related Adverse Events** | 26. Has your institution developed clinical alarm improvement initiatives over the past two years? | NO | Not sure | YES | — | — |
|  | 27. Does your institution have a program in place to improve the security of alarm management? | NO | Not sure | YES | — | — |
|  | ***28. Please fill in your opinion on " Alarm Related Adverse Events ":*** | | | | | |
| **Dimension VII Alert Related Adverse Events** | 29. Has your institution experienced adverse patient events in the last 2 years related to clinical alarm problems? | NO | Not sure | YES | — | — |
|  | 30. Since the implementation of the 2019 Patient Safety Goals issued by the Chinese Hospital Association regarding the alarm management program, your hospital has experienced a decrease in the occurrence of adverse events related to alarms. | Strongly disagree | Disagree | Not sure | Agree | Strongly disagree |
|  | ***31. Please fill in your opinion about "Alert Related Adverse Events ":*** | | | | | |

**Part Ⅲ** **Questionnaire on decision-making and decision-support regarding ECG alarms**

**[Introduction]:** There are 17 questions in this section, and the questionnaire covers three dimensions: confidence and difficulty in clinical decision-making, basis for decision-making, and need for decision support. Please answer the following questions according to the actual situation when you are working and choose the appropriate option, of which the 9th, 12th and 13th questions are multiple choice, and the rest is single choice.

**(Ⅰ) Confidence and Difficulty of Decision-Making**

**Confidence of decision-making about ECG alarms**

1. How confident are you in **setting thresholds for alarm parameters?**

□Low □Relatively Low □Not sure □Relatively High □High

1. How confident are you in **setting the alarm volume?**

□Low □Relatively Low □Not sure □Relatively High □High

1. How confident are you in the **timing of resetting or modifying alarm parameters?**

□Low □Relatively Low □Not sure □Relatively High □High

1. How confident are you in **handling alarms after they occur?**

□Low □Relatively Low □Not sure □Relatively High □High

**Difficulty of decision-making about ECG alarms**

1. How difficult are you in **setting thresholds for alarm parameters?**

□Low □Relatively Low □Not sure □Relatively High □High

1. How difficult are you in **setting the alarm volume?**

□Low □Relatively Low □Not sure □Relatively High □High

1. How difficult are you in the **timing of resetting or modifying alarm parameters?**

□Low □Relatively Low □Not sure □Relatively High □High

1. How difficult are you in **handling alarms after they occur?**

□Low □Relatively Low □Not sure □Relatively High □High

**(Ⅱ) Decision-Making Basis**

1. What are your primary bases for making decisions about ECG alarms?  **[Multiple choice]**

□Textbooks

□Colleagues and leaders

□Instruction books

□SOPs

□Clinical guidelines

□Systematic reviews

□Primary studies

□Thematic trainings

□Academic conferences

□Other

1. Please evaluate the effectiveness of the existing decision-making bases:

1) Textbooks:□Very Ineffective □Ineffective □Not sure □Effective □Very Effective

2) Colleagues and leaders:□Very Ineffective □Ineffective □Not sure □Effective □Very Effective

3) Instruction books:□Very Ineffective □Ineffective □Not sure □Effective □Very Effective

4) SOPs:□Very Ineffective □Ineffective □Not sure □Effective □Very Effective

5) Clinical guidelines:□Very Ineffective □Ineffective □Not sure □Effective □Very Effective

6) Systematic reviews:□Very Ineffective □Ineffective □Not sure □Effective □Very Effective

7) Primary studies:□Very Ineffective □Ineffective □Not sure □Effective □Very Effective

8) Thematic trainings:□Very Ineffective □Ineffective □Not sure □Effective □Very Effective

9) Academic conferences:□Very Ineffective □Ineffective □Not sure □Effective □Very Effective

10) Other:□Very Ineffective □Ineffective □Not sure □Effective □Very Effective

1. Please evaluate the accessibility of the existing decision-making bases:
2. Textbooks:□Very Inaccessible □Inaccessible □Not sure □Accessible □Very Accessible
3. Colleagues and leaders:□Very Inaccessible □Inaccessible □Not sure □Accessible □Very Accessible
4. Instruction books:□Very Inaccessible □Inaccessible □Not sure □Accessible □Very Accessible
5. SOPs:□Very Inaccessible □Inaccessible □Not sure □Accessible □Very Accessible
6. Clinical guidelines:□Very Inaccessible □Inaccessible □Not sure □Accessible □Very Accessible
7. Systematic reviews:□Very Inaccessible □Inaccessible □Not sure □Accessible □Very Accessible
8. Primary studies:□Very Inaccessible □Inaccessible □Not sure □Accessible □Very Accessible
9. Thematic trainings:□Very Inaccessible □Inaccessible □Not sure □Accessible □Very Accessible
10. Academic conferences:□Very Inaccessible □Inaccessible □Not sure □Accessible □Very Accessible
11. Other:□Very Inaccessible □Inaccessible □Not sure □Accessible □Very Accessible

**(Ⅲ) Decision support for ECG monitors**

1. If possible, which forms of decision support related to setting the parameters of ECG alarms would you prefer? **[Multiple choice question]**

□Intelligent Module Assisted Decision Support Systems

□Consultation with professionals

□Knowledge repository

□EBook

□Paper-based book

1. If possible, which forms of decision support related to handling alarms when they occurred would you prefer? **[Multiple choice question]**

□Intelligent Module Assisted Decision Support Systems

□Consultation with professionals

□Knowledge repository

□EBook

□Paper-based book

1. Do you think it is important to develop a standardized device interface to integrate ECG monitors with other devices?

□Not important □Not too important □Not sure □Important □Very important

1. Do you think it is important to develop an intelligent decision support for alarm threshold setting for newborns of different birth gestational ages module?

□Not important □Not too important □Not sure □Important □Very important

1. Do you think it is important to develop optimizing algorithm models to achieve accurate alarms?

□Not important □Not too important □Not sure □Important □Very important

1. Do you think it is important to develop Functional modules for grading alarms based on the severity of the patient's condition?

□Not important □Not too important □Not sure □Important □Very important
